# Psychodynamic Insights into Treatment-Resistant Pharmacotherapy: A Case Study Exploring Patient–Physician Dynamics and Adherence to Evidence-Based Practices

**DOI:** 10.3390/jpm14090897

**Published:** 2024-08-24

**Authors:** Alexander Baur, Leslie Kryzanowski

**Affiliations:** 1 Orthopaedics Department, Liberty University College of Osteopathic Medicine, Lynchburg, VA 24502, USA; 2 Virginia Beach Psychiatric Center, Virginia Beach, VA 23454, USA

**Keywords:** treatment resistance, psychodynamic pharmacology, behavioral pharmacology, insomnia, depression, anxiety, personality disorders

## Abstract

Background: Pharmacological resistance in severe recurrent mood and anxiety disorders remains a significant challenge in modern biological psychiatry. This case report investigates the intricate decision-making process employed by physicians when managing patients resistant to conventional pharmacotherapy. Methods: Informed consent was obtained from the patient. Following this, the case report was developed using the CARE checklist (2013) to ensure a comprehensive and systematic documentation of the treatment process and outcomes. Results: The patient’s treatment history highlights the complex nature of pharmacological resistance and the impact of minor medication adjustments versus established clinical practices. A crucial aspect of this case was the patient–physician relationship, particularly addressing the patient’s past grievances towards physicians, which played a significant role in the treatment process. Despite efforts to improve the physician’s confidence and approach, challenges such as lack of continuity and a fragile therapeutic relationship contributed to treatment failure. Conclusions: This case underscores the importance of psychodynamic models in overcoming pharmacologic challenges. A deeper understanding of the patient–physician dynamics and addressing underlying emotional factors can enhance treatment efficacy and patient outcomes, providing valuable lessons for managing complex cases of treatment resistance.

## 1. Introduction

In an era where an increasing number of individuals grappling with depression and anxiety seek solace in pharmacologic treatment, the inevitability of encountering treatment failure looms large. While some cases simply necessitate judicious adjustments to medications, leading to eventual stabilization, a unique subset of patients exhibit treatment failure due to psychodynamic aspects. Unraveling the complex psychodynamics using appropriate models is critical to connect the biologic psychiatry mindset and the psychoanalytical approach.

Beyond the conventional realm of pharmacological considerations, these instances demand a nuanced exploration of underlying causes contributing to treatment resistance. Thase and Rush created a five-stage strategy to properly identify and discuss treatment resistance [1]. In stages III and IV of resistance, they discuss how the therapeutic relationship is extremely valuable. In these very complex cases, the patients may exploit physician emotions through countertransference and dictate treatment. Delving deeper into this realm becomes imperative as it may reveal intricate therapeutic relationship dynamics, such as patients subconsciously transferring their anger to the physician, thereby undermining the prescribed treatment regimen.

The literature widely recognizes the advantages of combining pharmacotherapy and psychotherapy to optimize treatment outcomes [2]. Shapiro and Plakun were pioneers in using psychoanalytical dimensions to optimize pharmacologic treatment [3].

This case report centers on a patient displaying elevated resistance to conventional psychotropic medications, empowering them to influence treatment decisions and deviate from traditional evidence-based approaches. Our case report expands on the concept of psychodynamic psychopharmacology, which was described by Mintz and Belnap [4]. Additionally, the report delves into the influence of transference on physician confidence, highlighting the significance of a physician’s assurance in effectively managing challenging patients. Maintaining confidence is essential as it prevents the patient’s subconscious emotions from unintentionally steering the course of treatment, underscoring the need for a mindful and informed approach in such therapeutic scenarios.

## 2. Methods

This case report presents a 45-year-old woman with treatment-resistant anxiety and depression, shaped by complex psychodynamic factors. Informed consent was obtained, and the patient’s psychiatric history, treatment responses, and psychodynamic interactions were thoroughly documented. CARE guidelines were followed throughout to ensure a systematic and comprehensive review of the case. The analysis focused on the qualitative examination of the therapeutic relationship, particularly the impact of transference and countertransference on treatment outcomes.

Data collection involved a detailed review of medical records, psychiatric evaluations, and medication history, emphasizing the psychodynamic aspects of the patient’s condition. We drew upon the works of Gabbard to guide our psychodynamic analysis. Specifically, the psychodynamic model chosen for our case was object relation theory. Ethical considerations were strictly observed, with patient privacy and confidentiality protected throughout.

## 3. Patient Information

This case involves a 45-year-old married woman, currently unemployed, residing with her husband and children. She voluntarily admitted herself to a psychiatric hospital following presentation at a nearby emergency room, where she reported symptoms of anxiety and vague suicidal ideation. Of particular concern to the patient was her severely disrupted sleep, despite an extensive medication regimen. The patient met criteria for admission due to her suicidal ideation. The goal of inpatient treatment was to ensure patient safety and better control her acute psychologic symptoms.

Along with the suicidal thoughts, the patient reported generalized anxiety and occasional panic attacks marked by chest tightness. She reported an extensive history of insomnia due to the anxiety. However, she denied other panic attack symptoms such as shortness of breath, palpitations, a sense of doom, or tingling in her hands.

The patient revealed a history of a distressing childhood. The patient recalls her father being absent for much of her early childhood especially before she was 2 years old. Her mother served as the primary caregiver, while her father, a surgeon, was predominantly focused on his career. Thus, when her mother was diagnosed with amyotrophic lateral sclerosis (ALS) when she was 11 years old, it had a profound impact on her. The subsequent deterioration and eventual passing of her mother during the patient’s senior year of college had a substantial impact, leading to ongoing difficulties in fully comprehending and coping with this loss. Notably, there were no reports of emotional, physical, or sexual abuse, and the patient denied experiencing symptoms commonly associated with post-traumatic stress disorder (PTSD), such as nightmares or flashbacks.

Further exploration revealed a prolonged struggle with mental health challenges. While the patient experienced multiple traumatic events during childhood, she did not pursue mental health treatment until age 30. From the age of 30 until 44, the patient was consistently treated for anxiety and depression. The treatment during this 14-year period was self-reported as “fairly consistent”. The patient did report occasional changes in the specific selective serotonin reuptake inhibitors (SSRIs) and serotonin norepinephrine reuptake inhibitors (SNRIs) during this time. Before the age of 44, the patient found the most consistent benefit with paroxetine. The patient could not recall any specific stresses that could have preceded the onset of anxiety and depression. She reports having great relationships with her husband and children. However, she did not mention having any strong friends outside of her immediate family. It was unclear whether the patient had any social support outside of her husband.

Approximately a year ago, the patient was hospitalized for suicidal ideation. In the months leading up to this hospitalization, the patient found her medication regimen to be much less helpful. During the hospitalization she was stabilized and advised that her response to medications may change during the perimenopausal timeframe. In addition, the patient underwent psychological testing during this hospitalization and the patient was formally diagnosed with cluster B personality disorder with features of borderline and histrionic personality. Once discharged from the hospital she underwent nearly a year of intensive outpatient therapy without improvement. Of note, she refused dialectical therapy on multiple occasions in the outpatient setting. Thus, her outpatient therapy seemed to largely revolve around medication management rather than psychotherapy. The patient reported seeing her psychiatrist extensively over the past year. She was never able to go more than 6 weeks without an appointment to change the medication regimen. When asked about her experience with therapy, the patient was largely dismissive. On review of the last discharge, it was advised for her to complete a partial hospitalization program where she would have had group and individual therapy every weekday for 2 weeks. However, the patient refused and would only agree to seeing a therapist weekly. When questioned about her experience with this therapy, the patient was dismissive stating that she “tried for months and it did nothing”.

The patient’s medication management over the last 18 months had extreme fluctuations. Specifically, her anxiety and insomnia were not adequately controlled with more than three different classes of anti-depressants, including a tricyclic anti-depressant (TCA) and ketamine. All of these classes were taken for an adequate duration with proper dosing. She was unable to recall if she had ever taken a monoamine inhibitor (MAOi). She has never had ECT for her symptoms.

Her outpatient provider seemed to be at a loss after 12 months of medication changes, many of which were trials that did not follow evidence-based practices. Consequently, she was referred to a specialist on treatment resistance in the area. Before the appointment with the specialist, the patient felt extremely helpless and presented in the emergency room. In the ER, she was given Ativan for her anxiety and helplessness, but reported that this “made everything worse”. When questioned further, she was not able to describe how the lorazepam worsened her symptoms other than just repeating “it made me more anxious”.

During discussions about treatment options, a common pattern of dictating treatment and switching drugs also was evident. It was not evident whether the patient had a history of non-adherence or rather just was constantly frustrated with the medication regimen.

## 4. Diagnostic Assessment

Upon admission, a physical examination was conducted by a medical doctor, leading to diagnoses of hypertension and gasteroesophageal disease (GERD). These conditions were deemed adequately managed, and outpatient follow-up was recommended. Lab work, including complete blood count (CBC), complete metabolic panel (CMP), thyroid panel, lipid panel, and vitamin D levels, was also performed. Slightly elevated lipid levels were noted but did not require medication at this time. A deficiency in vitamin D was identified with a level of 20.7, prompting the initiation of vitamin D replacement. All other lab results were within normal limits.

The previous psychological testing from an earlier hospitalization was reviewed. The DSM-5-based diagnoses included generalized anxiety disorder, panic disorder, recurrent severe major depressive disorder without psychosis, moderate benzodiazepine use disorder, and a cluster B personality disorder with borderline and histrionic features.

A psychiatrist evaluated the patient upon admission and confirmed the diagnoses of generalized anxiety disorder, recurrent moderate major depressive disorder without psychosis, and moderate benzodiazepine use disorder. While the history of cluster B personality disorder and panic disorder was noted, these were not initially apparent during the interview.

Daily evaluations throughout the hospitalization revealed a more pronounced presence of cluster B personality disorder, particularly with borderline features such as splitting. The severity of benzodiazepine use disorder was reassessed as severe rather than moderate. After a week of inpatient treatment, the prognosis was extremely poor due to significant issues with insight and adherence. Resistance to individual psychotherapy emerged as a major barrier to successful treatment.

## 5. Hospital Course

The objective of inpatient treatment was to stabilize the patient, address suicidal ideations, and alleviate feelings of helplessness. Additionally, hospitalization aimed to reduce acute symptoms and provide guidance for her subsequent outpatient care. Notably, due to her extensive psychiatric history, the prognosis of completely resolving her symptoms during this hospitalization was poor.

The patient’s history of extensive psychotropic medication trials guided the selection of the most appropriate regimen. She was unwilling to start with any evidence-based medications that she had previously deemed unsuccessful. She was started on imipramine due to refusing all SSRIs and SNRIs. Additionally, she had been discharged on imipramine at her last hospitalization with positive response. Along with the imipramine, gabapentin and mirtazapine were deemed suitable to help with her sleep and anxiety.

Daily interactions with the patient primarily revolved around medication discussions as she was very resistant to any therapy. The patient consistently expressed dissatisfaction and pressured the physician for changes well before the therapeutic benefits could be thoroughly assessed. This persistent push for alterations is reflected in Table 1, illustrating extensive deviations from standard clinical practice. Subsequently, the patient was exposed to many more side effects than if a consistent medication regimen had been properly adhered to.

The therapeutic relationship and physician behavior, particularly countertransference, were clear during this hospitalization. A thorough interview revealed a difficult upbringing contributing to a personality disorder and medication resistance, emphasizing unresolved anger towards her absent father, who was a physician, and suppressed memories.

Post-interview, it was clear that dialectical therapy would benefit the patient; however, she was very resistant to anything other than medications, as seen in her prior refusals of dialectical outpatient therapy. She refused to attend group and individualized therapy. On the 8th day, a suitable long-term inpatient facility was found, emphasizing psychotherapy. Despite some progress and the physician identifying what the patient truly needed, the patient sought another doctor, resumed clonazepam, and refused the medications that had contributed to her slight improvement. The weak therapeutic relationship was detrimental in this case because the physician was not able to provide the best care for this patient. Over the week there were slight improvements, but the patient’s self-sabotaging behavior ultimately was not able to be overcome.

The course of treatment underscores the interplay of psychotropic medications, patient autonomy, and the therapeutic relationship. Comprehensive approaches considering both pharmacological and psychoanalytical dimensions are crucial. The patient’s recovery ultimately hinges on securing proficient professionals who guide treatment confidently, avoiding patient-driven decisions for successful outcomes.

This highlights the necessity for a comprehensive approach that takes into account physician emotions in medication management. The extensive medication changes illustrated in Table 1 show how the patient dictated treatment and deviated from standard practice. This unique situation shows how a lack of continuity in care can be detrimental to treatment.

## 6. Follow-Up and Outcomes

Throughout the hospitalization, the patient displayed poor adherence and tolerability to medications. Multiple adverse events, including severe suicidal ideation and dissatisfaction with the psychiatrist, were reported. Despite a week of inpatient management and numerous medication changes, the patient’s symptoms remained largely unchanged. Clinical improvement was minimal, and the prognosis for this patient is deemed extremely unfavorable due to lack of insight, poor judgment, and resistance to psychotherapy. The weak therapeutic relationship and excessive polypharmacy, and not following evidence-based guidelines, contributed to the overall poor outcomes. The patient’s resistance to treatment and the impact of physician emotions on decision-making are evident in this challenging case.

## 7. Discussion

The patient’s trauma and overreliance on concrete means to manage internal distress had a significant impact of the pharmacologic effectiveness in this case. Specifically, her own fantasies of what medications would do for her influenced her requests and impacted the physician’s management. Additionally, this case report underscores the profound influence of physician emotions on medication management. In an ideal scenario, physicians should make decisions impartially, but a lack of confidence may lead them toward the path of least resistance, jeopardizing the best interests of the patient. Straying from evidence-based clinical practices exposes patients to considerable side effects without apparent benefits, as highlighted by the significant adverse effects our patient encountered during hospitalization. Not adhering to evidence-based practice also may present as a pseudo-resistance. Additionally, the patient’s non-compliance could compound a pseudo-resistance [5]. A nuanced comprehension of the patient–physician relationship is essential for adeptly addressing challenging cases characterized by treatment resistance, especially in the later stages described by Thase and Rush [1].

One noteworthy takeaway from this case report is the importance of a resilient physician who acknowledges their own emotions and avoids yielding to the patient’s preferences. Furthermore, maintaining continuity in care is imperative for this subset of patients. Given the complexity of their psychiatric history and underlying symptoms, a substantial amount of time is required to gain a comprehensive understanding and provide optimal treatment. Even with significant advancements in psychiatric pharmacology, this case report highlights how a biological framework is not always effective. Vlastelica emphasizes the importance of identifying dynamic factors that may be interfering in pharmacologic treatment [6].

This specific scenario aligns with the literature on the psychodynamics of psychopharmacology. Mintz and Belnap were pioneers in this field and discuss how the use of many psychodynamic models can have a significant impact on pharmacologic benefit [4]. They found that an understanding beyond the biologic perspective increases overall clinical effectiveness of the medication. Building on this concept, the paper by Silvio and Condemarin explores how, over the past 20 years, there has been an increased focus on how incorporating medications can affect individuals psychologically [7]. In their paper, they recognize the importance of interpersonal factors in patient adherence and ultimate success. A similar study by Li confirmed the importance of being mindful of various psychodynamic models and utilizing them in clinical practice in conjunction with medications [8].

In this case, unaddressed psychodynamic factors rooted in object relations theory likely played a crucial role. According to the theory first described by Klein, early relationships, internalized during infancy, continue to shape interpersonal dynamics throughout life [9]. The patient may have struggled with transitioning from Klein’s “paranoid-schizoid position”—a state characterized by splitting the world into “good” and “bad” objects—to the “depressive position,” where an integration of these split elements occurs [10]. This failure to integrate could have led to the patient projecting hostility onto the medical team, fueled by primitive anxieties or a fear of psychological disintegration. Such dynamics, if unrecognized, may have exacerbated the patient’s resistance to treatment. Addressing these underlying psychodynamic issues within the therapeutic relationship might have alleviated some of the patient’s hostility and improved the overall treatment outcome. A common theme within the realm of psychodynamics of psychopharmacology is the therapeutic alliance. This is a concept that has been researched extensively and is agreed to be integral in the success of treatment. The meta-analysis by Martin et al. in 2000 showed the significance of a positive therapeutic relationship [11]. Taking these concepts and applying them in clinical practice can be a challenge. The book by Reba and Balon focuses on combining pharmacotherapy and psychotherapy [12]. They emphasize the importance of a comprehensive initial diagnostic assessment. In practice, a comprehensive assessment can be extremely challenging when patients are resistant, as in our case report. The concepts in their book were confirmed by the meta-analysis performed by Karyotaki et al. in 2016 [13]. Nonetheless, it is important to still understand the psychodynamic aspects even if psychotherapy is not an option for treatment. For example, Forrest talks about how being aware of certain character styles can improve the therapeutic alliance and medication regimen [14].

We can delve deeper into the specific nuances that contributed to the poor therapeutic relationship in our case report. Psychodynamic formulation was first described by Perry et al., which focuses on central conflicts such as transferences and resistances [15]. Their paper discusses how psychodynamic formulation is important in guiding psychiatric treatment. Transference is a prominent coping mechanism used by the patient. Her subconscious deep-seated anger towards her father was transferred to the physicians treating her. Therefore, multiple physicians proceeded to deviate from traditional practice and made excessive medication changes due to the patient’s demands. These excessive medication changes were also likely compounded by the physicians feeling helpless themselves from the patient’s countertransference of her emotions.

Transference, initially introduced by Freud and further developed by Carl Rogers, has evolved and been applied to the therapeutic alliance. A comprehensive review of transference by Horvath in 2000 explores its current implications in clinical practice [16]. The primary takeaway emphasizes the critical importance of identifying transference early in the therapeutic relationship and recognizing the collaborative framework’s significance in determining the most effective therapy [2]. Similar papers by Marcus in 2007 and Gabbard in 2020 echoed many of the same concepts but delved deeper into how physicians should use their emotions to better understand their patient’s subconscious [17,18]. The paper by Marcus specifically explored the transference and countertransference related to medications. He concluded that both of these ego defenses are highly specific diagnostic indicators [18]. While this case report was not successful in treating the patient, the identification of countertransference was used to understand the underlying emotions and create a plan for a future physician to follow. This unique case underscores the impact of subconscious emotions on the success of treatment in patients with underlying personality disorders.

Our case report underscores the importance of integrating psychoanalytic and pharmacological approaches in the treatment of borderline personality disorder. A significant factor in the therapeutic failure was the patient’s refusal to engage in cognitive behavioral therapy (CBT) or dialectical behavior therapy (DBT). The patient exhibited a pronounced splitting mechanism, categorizing physicians as either idealized figures who acceded to her demands or devalued figures when they prescribed medications associated with adverse effects. This splitting behavior, characterized by cycles of idealization and devaluation, severely disrupted the therapeutic alliance. Each time alternative treatment modalities were proposed, the patient exhibited marked distress, further complicating her clinical management and contributing to the overall therapeutic impasse.

If CBT or DBT cannot be accomplished, understanding the principles can still be beneficial in optimizing the pharmacological treatment. To understand how to apply these concepts to management, we can draw upon the “A View from Riggs” publication series, particularly focusing on the psychodynamic approach to understanding treatment resistance. In Plakun’s foundational paper, he underscores the necessity of tolerating negative transference as a frequent component associated with treatment resistance [19]. Plakun argues that recognizing the provider’s own negative emotions in countertransference is crucial. Furthermore, the paper highlights the importance of not relinquishing authority to the patient, emphasizing the need for maintaining control over treatment strategies and admission terms.

In another publication from the same series, Shapiro delves into the dynamics of the patient’s living situation and authority [3]. The paper highlights the risk of physicians adhering solely to the current treatment paradigm, neglecting the individual’s personality and psyche. Without a comprehensive psychological understanding, biological interventions offer limited benefits. For these patients, resistance to treatment may not only be a reenactment of painful experiences, but also a mode of communication. Their resistance may be a coping mechanism for suppressed anger, allowing them to assert control over providers they deem untrustworthy.

Both our case report and the “A View from Riggs” publication series exemplify how recognizing individuals’ subconscious psychodynamics can transform physicians into competent allies, leading to a shift in their own perspectives.

Our case report highlights the inherent challenges in managing patients with complex medical conditions. When a therapeutic alliance is weak, the repercussions of excessive polypharmacy become particularly pronounced. In such instances, clinical pharmacologists play a pivotal role as a crucial safeguard. The study conducted by Stuhec and Zorjan underscores the significance of an external perspective in evaluating reported benefits and clinical relevance within a specified timeframe [20].

Clinical pharmacists, as demonstrated in their interventions with ambulatory psychogeriatric patients, contribute valuable insights to the decision-making process. Their specialized knowledge enables a more comprehensive and well-informed approach to combining different medications [20]. This collaborative strategy not only adds an additional layer of scrutiny to medication choices, but also serves to counterbalance the potential influence of physician emotions on decision-making. The outcome is a more objective and patient-centered approach to care.

## 8. Conclusions

In conclusion, the optimal care for challenging cases necessitates the integration of psychopharmacological and psychodynamic models. Recognizing and addressing transference and splitting mechanisms early in the therapeutic relationship are crucial to successful treatment. This case report underscores the active role of patients in influencing treatment decisions. To mitigate variance from evidence-based practice, physicians must confidently navigate these dynamics. A strong therapeutic relationship and a multidisciplinary approach are pivotal for the proper management of these unique patients. Therefore, adopting a holistic approach that considers both pharmacological and psychoanalytical dimensions is essential for ensuring comprehensive and effective care in challenging cases.

## Figures and Tables

**Table 1:** Summarized hospital course.

Day	Patient Events	Medication Changes	Dose Changes	Patient Status
0	Required clonazepam at night	Imipramine Gabapentin Mirtazapine		Poor
1	TCA made her suicidal Learned never fully stopped benzo use Refused to take benzo Received psychotherapy		Dec imipramine Inc gabapentin	Poor
2	Wanted to see another doctor Passive aggressive towards doctor Required many PRNs ○ Diphenhydramine ○ Trazadone ○ Propranolol		Inc gabapentin Inc mirtazapine	Very poor
3	Called outpatient provider No longer experiencing depression Focused on insomnia	2 changes D/C imipramine Start nortriptyline		Improvement
4	Experiencing more depression	4 changes D/C mirtazapine Start propranolol Start quetiapine Start ramelteon		Stable
5	Stated she “disliked all the medication” Hypotensive Slept 7 h	1 change D/C ramelteon	Inc quetiapine	Significant improvement
6	Patient appears more rested with improved mood and affect States “feels weird”	1 change D/C propranolol	Inc gabapentin Inc quetiapine	Poor
7	Switched doctor New doctor restarted clonazepam	4 changes D/C nortriptyline D/C gabapentin Start lithium Start clonazepam		Very poor

D/C: Discontinue, TCA: Tricyclic acid, Benzo: benzodiazepine, PRN: As needed medications.

## Data Availability

The data supporting this case report are available upon request, subject to Institutional Review Board (IRB) approval. Requests for data access should be directed to the corresponding author at alexbaur123@gmail.com. Data will be de-identified to ensure patient privacy. Access is granted for research purposes only, pending IRB approval and compliance with ethical guidelines.
